# Open label study of ambrisentan in patients with exercise pulmonary hypertension

**DOI:** 10.1177/2045893217709024

**Published:** 2017-05-12

**Authors:** Sergio A. Segrera, Laurie Lawler, Alexander R. Opotowsky, David Systrom, Aaron B. Waxman

**Affiliations:** Center for Pulmonary-Heart Diseases, Pulmonary Vascular Disease Program, Pulmonary Critical Care Medicine, Brigham and Women's Hospital, Boston, MA, USA

**Keywords:** cardiopulmonary exercise testing (CPET), dyspnea, exercise, pulmonary hypertension, treatment

## Abstract

A growing body of evidence suggests that exercise pulmonary hypertension (ePH) is an early form of pulmonary arterial hypertension (PAH). Identifying the disease at an early, potentially more responsive phase, and initiating treatment may improve functional status and prevent progression to severe forms of PAH. This was a single-center, open-label six-month treatment trial to evaluate the effect of ambrisentan on pulmonary hemodynamics and exercise capacity in ePH utilizing invasive cardiopulmonary exercise testing (iCPET). After six months of treatment with ambrisentan, patients repeated iCPET; exercise capacity, symptoms, and pulmonary hemodynamics were reassessed. Twenty-two of 30 patients completed the treatment phase and repeat iCPET. After six months of treatment there was a significant decline in peak exercise mPAP (−5.2 ± 5.6 mmHg, *P* = 0.001), TPG (−7.1 ± 8.0 mmHg, *P* = 0.001), PVR (−0.9 ± 0.7 Woods units, *P* = 0.0002), and Ca-vO_2_ (−1.8 ± 2.3 mL/dL, *P* = 0.0002), with significant increases in peak PCWP (+2.9 ± 5.6 mmHg, *P* = 0.02), PVC (+0.8 ± 1.4 mL/mmHg, *P* = 0.03), and CO (+2.3 ± 1.4 L/min, *P* = 0.0001). A trend toward increased VO_2_max (+4.4 ± 2.6% predicted, *P* = 0.07) was observed. In addition, there were improvements in 6MWD and WHO FC after 24 weeks. Our findings suggest that treatment of ePH with ambrisentan results in improved pulmonary hemodynamics and functional status over a six-month period. Treatment of ePH may prevent the progression of vascular remodeling and development of established PAH.

## Introduction

Exercise pulmonary hypertension (ePH) remains a controversial topic among experts in pulmonary vascular disease, and was omitted from the clinical classification of pulmonary hypertension in 2008^1^ and 2013^2^ due to a lack of universally accepted upper limits of normal for exercise pulmonary hemodynamics. We have previously characterized ePH in a large group of symptomatic patients using direct measurements of pulmonary hemodynamics at rest and during maximum incremental cardiopulmonary exercise testing.^3^ The pattern and severity of the pulmonary hemodynamic response in ePH is intermediate between that of normal participants and patients with resting pulmonary arterial hypertension (PAH),^3^ and is associated with reduced exercise capacity and decreased quality of life.^4^ Similar to what is seen in World Health Organization (WHO) Group-1 PAH, patients with ePH have right ventricular-pulmonary vascular uncoupling^3^ and a metabolic profile consistent with a nitric oxide deficient state.^5^ Recent studies further support the hypothesis that ePH represents a mild, early phase of PAH.^6–10^

While significant strides have been made in the development of treatment of PAH, prognosis remains poor.^11^ On average, patients are symptomatic for more than two years before a diagnosis of PH is made.^12^ By the time of diagnosis, there is already extensive remodeling of the pulmonary vascular bed. As studies have demonstrated, borderline resting or elevated pulmonary arterial pressures during exercise may predict the development of PAH.^13–18^ Consequently, screening and early detection of ePH might identify a group of patients more responsive to treatment aimed at preventing progression to resting PAH. Few studies have investigated the treatment of ePH patients.^19–22^ With such a paucity of data, additional studies of treatment of ePH are warranted.

Ambrisentan is a propanoic acid class, A-selective, high affinity endothelin receptor antagonist (ERA) that has demonstrated significant improvement in six-minute walk distance (6MWD) in Phase 2 and Phase 3 efficacy studies in patients with PAH.^23^ Clinically meaningful improvements were also seen for Borg Dyspnea Score, WHO functional class (FC), quality of life, and cardiopulmonary hemodynamics, with a low incidence of relevant serum aminotransferase abnormalities.^23,24^ In addition, the drug’s half-life allows for once-daily dosing, a characteristic associated with improved participant compliance.

We sought to evaluate the effects of ambrisentan in patients with ePH administered orally for six months on pulmonary hemodynamics and exercise capacity utilizing invasive cardiopulmonary exercise testing (iCPET). Secondary objectives include the effect of ambrisentan treatment on improvements in 6MWD and WHO FC.

## Methods

### Design and study population

This was a single-center, open-label, uncontrolled treatment trial of of ePH with the pulmonary vasodilator ambrisentan. We identified 30 consecutive adults with a newly confirmed diagnosis of clinically stable ePH, not previously treated with any pulmonary vasodilator, and without any clinically significant co-morbidities (see online supplement for full details of inclusion/exclusion criteria and study protocol). Throughout the six-month treatment phase, no patient was treated with another pulmonary vasodilator, enrolled in cardiac or pulmonary rehabilitation, or a formal exercise training program of any kind.

Partners Human Research Committee approved this study (protocol 2008P000687, NCT01338636). All patients were recruited after having had a clinically indicated iCPET performed for the purpose of evaluating unexplained exertional intolerance. Patients were considered for the study if they were aged over 18 years, had findings of ePH on an iCPET performed within the six months prior to entry into the study. Participants provided written informed consent prior to any study-related procedures or assessments. ePH was defined as mean pulmonary artery pressure (mPAP) of > 30 mmHg, pulmonary capillary wedge pressure (PCWP) of < 20 mmHg, and pulmonary vascular resistance (PVR) of > 1 Woods units (WU) at peak exercise and in the absence of resting PAH.^3^ Female participants of childbearing potential were required to use a minimum of two forms of contraceptive therapy, including at least one barrier method. In all cases, concomitant medications were stable for at least four weeks prior to enrollment in the study and did not change during the study period and follow-up. Changes in diuretic therapy were made as needed during the study period.

Once enrolled, baseline WHO FC, 6MWD, and Borg Dyspnea Score were assessed. Immediately after all baseline measurements were obtained, ambrisentan therapy was initiated at 5 mg by mouth daily. All patients were evaluated at weeks 4, 8, 12, 16, and 20, during which 6MWD and Borg Dyspnea Score, WHO FC, concomitant medications, and adverse events were assessed. At the week 4 visit, patients tolerating the 5 mg dose were increased to 10 mg for the duration of the study. At the end of the six-month treatment phase, participants underwent a repeat iCPET.

The iCPET methodology has been described elsewhere.^25,26^ Briefly, simultaneous measurements of ventilation, breath-by-breath respiratory gas exchange, arterial and mixed venous blood gas sampling, and pulmonary hemodynamics are assessed at rest and during incremental upright cycling to exhaustion.

### Statistical analysis

Continuous variables are expressed as mean ± standard deviation, while categorical variables are reported as the percentage of patients. Data from the treatment phase were compared to baseline for all efficacy endpoints. If the variable was normally distributed, a paired *t*-test was used to compare values; the Wilcoxon signed rank test was used for non-normally distributed data. All statistical analyses were performed using Stata 14 (StataCorp LP, College Station, TX, USA) and SPSS 23 (IBM Corp, Armonk, NY, USA).

## Results

Thirty patients with ePH met inclusion criteria and agreed to participate in the study. Of these, 22 completed the six-month treatment phase and underwent a repeat iCPET. Baseline characteristics of these study patients are presented in Table 1. The majority of patients were women (63.6%) and white (95.5%), with an average age of 58.6 ± 9.9 years. In all cases co-morbidities (listed in Table 1) were considered not clinically significant based on objective testing that showed mild changes according to predetermined inclusion criteria (see online supplement). Participants had a mild limitation of physical activity at baseline (mean WHO FC 2 ± 0.6).

**Table 1.** Baseline characteristics of study participants (n = 22).

Age (years)	58.6 ± 9.9
Men (n (%))	8 (36.4)
Women (n (%))	14 (63.6)
Race (n (%))	
White	21 (95.5)
African American	1 (4.5)
Ethnicity (n (%))	
Not Hispanic/Latino	22 (100)
Associated co-morbidities (n (%)	
COPD	5 (22.7)
OSA	4 (18.2)
ILD	3 (13.6)
CTD	5 (22.7)
VTE	6 (27.3)
WHO FC	2 ± 0.6
6MWT	
Distance walked (m)	402.4 ± 130.6
Borg Dyspnea Score	5.1 ± 2.6

**Table 2.** Baseline characteristics of participants withdrawn (n = 8).

Age (years)	
Men (n (%))	3 (37.5)
Women (n (%))	5 (62.5)
Race (n (%))	
White	8 (100.0)
Ethnicity (n (%))	
Not Hispanic/Latino	8 (100)
Associated co-morbidities (n (%))	
COPD	1 (12.5)
OSA	3 (37.5)
CTD	2 (25.0)
VTE	3 (37.5)
WHO FC	
6MWT	
Distance walked (m)	390.3 ± 106.0
Borg Dyspnea Score	6.9 ± 2.7
Resting hemodynamics	
mPAP (mmHg)	18.0 ± 3.6
PCWP (mmHg)	7.4 ± 3.0
TPG (mmHg)	10.9 ± 3.0
PVR (WU)	2.7 ± 1.0
CO (L/min)	4.3 ± 0.7
Ca-vO2 (mL/dL)	8.0 ± 1.3
Peak exercise hemodynamics	
mPAP (mmHg)	36.5 ± 4.0
PCWP (mmHg)	13.1 ± 4.6
TPG (mmHg)	23.4 ± 3.4
PVR (WU)	2.2 ± 0.7
PVC (mL/mmHg)	3.6 ± 1.4
CO (L/min)	11.45 ± 2.6
VO2max (% predicted)	68.8 ± 23.4
Ca-vO2 (mL/dL)	10.9 ± 2.0
VE/VCO2 slope	32.0 ± 9.2
VO2 at AT (mL/min)	837.7 ± 285.5

WHO FC, World Health Organization functional class; 6MWT, six-minute walk test; mPAP, mean pulmonary artery pressure; PCWP, pulmonary capillary wedge pressure; TPG, transpulmonary pressure gradient; PVR, pulmonary vascular resistance; PVC, pulmonary vascular compliance; CO, cardiac output; VO2max, maximum oxygen uptake; Ca-vO2, arteriovenous oxygen content difference; VE/VCO2, minute ventilation/carbon dioxide production; VO2 at AT, oxygen consumption at anaerobic threshold.

Resting pulmonary hemodynamics at baseline and week 24 are presented in Table 3. No significant changes in resting hemodynamics were evident between baseline and study end. However, there was a trend toward increased resting cardiac output (CO) (*P* = 0.08).

**Table 3.** Resting hemodynamics (n = 22).

	Baseline	Week 24	*P*
mPAP (mmHg)	17.5 ± 4.2	17.0 ± 2.8	0.5
PCWP (mmHg)	8.3 ± 3.3	7.9 ± 3.3	0.5
TPG (mmHg)	9.3 ± 2.8	9.1 ± 2.8	0.7
PVR (WU)	1.94 ± 0.8	1.8 ± 0.8	0.3
CO (L/min)	5.1 ± 1.4	5.8 ± 1.9	0.08
Ca-vO_2_ (mL/dL)	6.7 ± 0.9	6.4 ± 1.4	0.5

mPAP, mean pulmonary artery pressure; PCWP, pulmonary capillary wedge pressure; TPG, transpulmonary pressure gradient; PVR, pulmonary vascular resistance; CO, cardiac output; Ca-vO_2_, arteriovenous oxygen content difference.

**Fig. 1.**
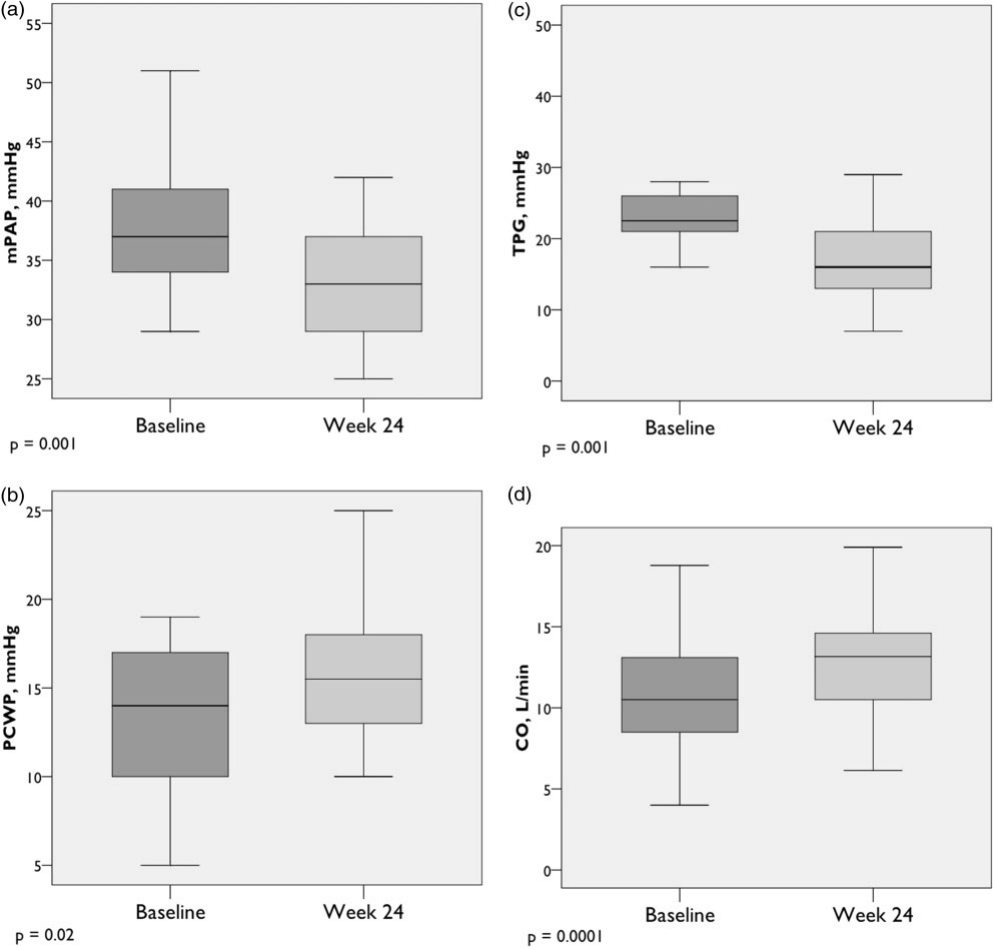
Peak exercise hemodynamics (n = 22). (a) Mean pulmonary artery pressure; (b) pulmonary capillary wedge pressure; (c) transpulmonary pressure gradient; (d) cardiac output. Box plots show median, IQR, minimum, and maximum values (± 1.5·IQR).

**Fig. 2.**
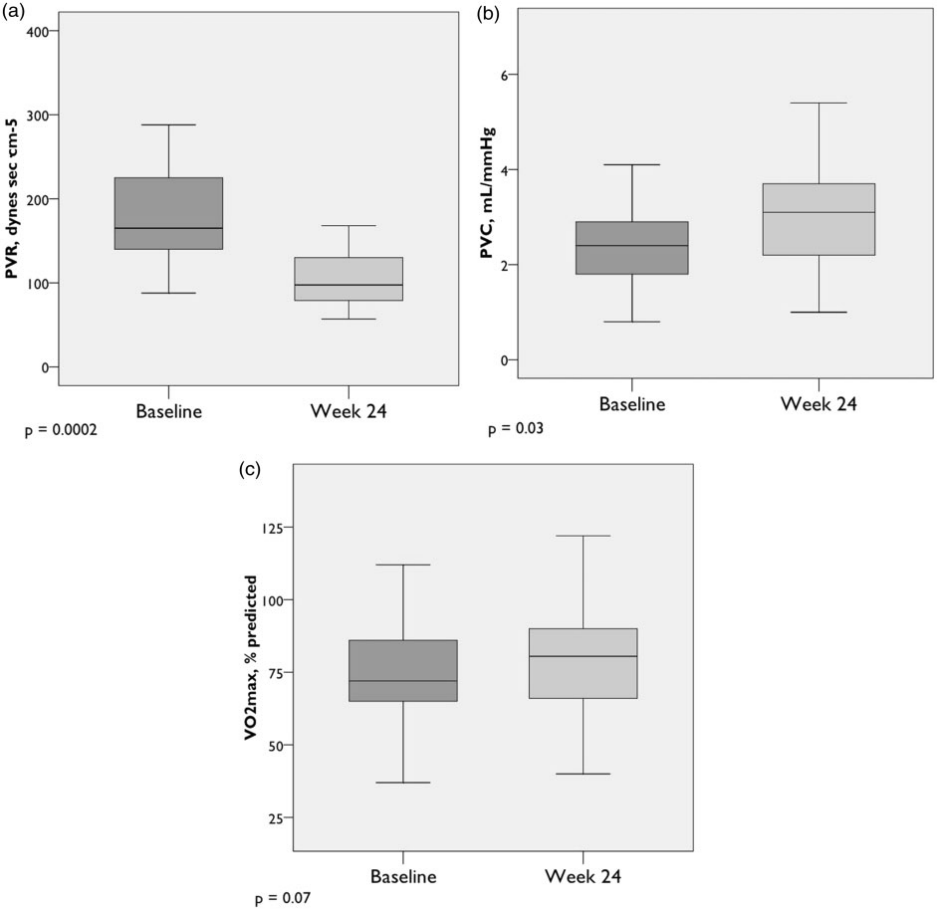
Peak exercise hemodynamics (cont.) (n = 22). (a) PVR = Pulmonary vascular resistance; (b) Pulmonary vascular compliance; (c) Maximum oxygen uptake. Box plots show median, IQR, minimum, and maximum values (±1.5·IQR).

Peak exercise pulmonary hemodynamics improved significantly from baseline to week 24. On average, exercise hemodynamics normalized (Table 4, Figs. 1 and 2). Notably, there was a significant decline in peak mPAP (−5.2 ± 5.6 mmHg, *P* = 0.001), transpulmonary pressure gradient (TPG) (−7.1 ± 8.0 mmHg, *P* = 0.001), and PVR (−0.9 ± 0.7 WU, *P* = 0.0002), with significant increases in peak pulmonary vascular compliance (PVC) (+0.8 ± 1.4 mL/mmHg, *P* = 0.03) and CO (+2.3 ± 1.4 L/min, *P* = 0.0001). A trend toward increased maximum oxygen uptake (VO_2_max) (+4.4 ± 2.6% predicted, *P* = 0.07) was observed. Interestingly, after six months of treatment, while there was no change in resting PCWP, there was a statistically significant increase in peak exercise PCWP compared to baseline (+2.9 ± 5.6 mmHg, *P* = 0.02). In spite of the increase, the peak exercise PCWP remained well within normal peak exercise limits. There were no significant changes in ventilatory efficiency (VE/VCO_2_ slope) or VO_2_ at the anaerobic threshold (AT) after 24 weeks.

**Table 4.** Peak exercise hemodynamics (n = 22).

	Baseline	Week 24	*P*
mPAP (mmHg)	38.0 ± 6.6	32.8 ± 5.2	0.001
PCWP (mmHg)	12.9 ± 4.2	15.7 ± 4.2	0.02
TPG (mmHg)	24.4 ± 7.0	17.2 ± 5.6	0.001
PVR (WU)	2.3 ± 0.9	1.4 ± 0.5	0.0002
PVC (mL/mmHg)	2.5 ± 0.9	3.3 ± 1.4	0.03
CO (L/min)	10.9 ± 3.8	13.2 ± 3.8	0.0001
VO_2_max (% predicted)	75.0 ± 19.3	79.4 ± 21.4	0.07
Ca-vO_2_ (mL/dL)	12.5 ± 2.3	11.1 ± 2.3	0.0001
V_E_/VCO_2_ slope	33.3 ± 7.5	33.6 ± 6.6	0.76
VO_2_ at AT (mL/min)	838.3 ± 365.9	874.3 ± 375.2	0.94

mPAP, mean pulmonary artery pressure; PCWP, pulmonary capillary wedge pressure; TPG, transpulmonary pressure gradient; PVR, pulmonary vascular resistance; PVC, pulmonary vascular compliance; CO, cardiac output; VO_2_max, maximum oxygen uptake; Ca-vO_2_, arteriovenous oxygen content difference; V_E_/VCO_2_, minute ventilation/carbon dioxide production; VO_2_ at AT, oxygen consumption at anaerobic threshold.

Among the secondary endpoints, 6MWD improved by 34.8 ± 73.2 m (*P* = 0.04) after 24 weeks of treatment. In addition, perceived dyspnea during the six-minute walk test (6MWT) improved, from a baseline Borg Dyspnea Score of 5.1 ± 2.8 (intense breathlessness) to 3.0 ± 0.9 (moderate breathlessness) after 24 weeks (*P* = 0.002). A significant decrease in WHO FC was also observed, from 2.0 ± 0.6 at baseline to 1.5 ± 0.6 at study end (*P* = 0.002). Improvements in exercise capacity and FC were noticed by every patient at month 3.

Adverse events seen during the 24-week treatment with ambrisentan are presented in Table 5. Common adverse events included peripheral edema, nasal congestion, headache, flushing, and increased cough. These adverse reactions have been reported previously in clinical trials of ambrisentan and were anticipated. Less common events included joint and body pain, rash, upper respiratory infection, and gastrointestinal infection. Jaw pain, nose bleed, palpitations, and dizziness rarely occurred. All adverse drug reactions were mild to moderate and resolved spontaneously during the study or were well tolerated.

**Table 5.** Adverse events.*

Peripheral edema	7
Nasal congestion	7
Headache	5
Flushing	4
Increased cough	4
Joint and body pain	3
Rash	2
Upper respiratory infection	2
Gastrointestinal infection	2
Jaw pain	1
Nose bleed	1
Palpitations	1
Dizziness	1
Worsening dyspnea on exertion^†^	8

* Total number of events; in some participants who completed study >1 event occurred.

† Participants withdrawn from study.

Eight participants failed to improve during treatment with ambrisentan and experienced subjective lack of improvement or worsening dyspnea on exertion. These participants were subsequently withdrawn from the study, between the week 4 and week 8 visits. Baseline characteristics of participants who were withdrawn from study, including resting and peak exercise hemodynamics, are presented in Table 2. End-of-study measurements of exercise hemodynamics were not assessed in these patients. Mean 6MWD of the last 6MWT carried forward was not significantly different from baseline (−21.1 ± 72.0 mmHg, *P* = 0.4). Five of the eight participants withdrawn were transitioned to a PDE-5 inhibitor for treatment of ePH. All five noted subjective improvement of their dyspnea on exertion, and demonstrated improved exercise capacity based on either 6MWD or submaximum exercise testing. Three of the eight participants who were withdrawn from the study were lost to follow-up.

## Discussion

Our results suggest that treatment of ePH with ambrisentan is well tolerated among most and is associated with significant improvement of exercise pulmonary hemodynamics, symptoms, and functional status. Few studies have investigated the treatment of ePH patients. An open-label pilot study of the use of ambrisentan in 12 patients with ePH associated with systemic sclerosis (SSc) demonstrated an improvement in exercise hemodynamics and 6MWD over 24 weeks, with significant changes in exercise PVR, mPAP, and CO.^19^ Park et al. found a significant increase in 6MWD among a small cohort ten patients after short-term (median 189 days) and long-term (median 416 days) treatment with bosentan or sildenafil.^20^ Two additional pilot studies among patients with SSc-associated ePH showed improvements in hemodynamic parameters after treatment with bosentan.^21,22^ Ours is the first study to demonstrate the extended benefit of treatment of ePH with an ERA marked by an improved pulmonary vascular response to exercise.

In PAH, intimal proliferation and fibrosis, medial hypertrophy, and in situ thrombosis characterize the pathological findings in the pulmonary vasculature.^27^ At earlier stages, vascular remodeling may be confined to the small pulmonary arteries, resulting in decreased compliance of the pulmonary vasculature. As pathologic remodeling progresses, PVR and right ventricular work increase, along with a corresponding increase in mPAP. Among patients with ePH, our study showed significant improvements of mPAP, PVR and PVC, and 6MWD and dyspnea after 24 weeks of treatment with ambrisentan, which may reflect prevention of progression of resting PAH.

Pulmonary vascular compliance, calculated as stroke volume over pulmonary arterial pulse pressure (SV/PP), is an accepted estimate of the total arterial compliance of the pulmonary vascular tree and reflects the ability of the vascular bed to distend in response to RV contraction and recoil during diastole.^28^ Unlike PVR, which quantifies flow and resistance in the distal pulmonary arterial vascular bed, PVC reflects the elastic properties that modulate the impact of pulsatile blood flow. Decreased pulmonary arterial compliance and distensibility may represent early dysfunction and remodeling of the pulmonary vascular bed.^29^ Previously, we found that PVC was reduced at rest, peak exercise and throughout recovery in ePH,^10^ indicating a more pronounced and sustained pulmonary vascular stiffness compared to normal controls and those with exercise HFpEF. In the present study, a significant decrease in resistance was accompanied by a significant increase in compliance after six months of treatment. Interestingly, in 11 patients we evaluated PVC during recovery from peak exercise and found that the compliance recovery pattern had normalized. Taken together, these findings, at peak exercise and during recovery, could be attributed to vasodilation, or could suggest reverse remodeling of the distal pulmonary vascular bed in patients with ePH.

Patients with hemodynamic evidence of left ventricular diastolic dysfunction were excluded from the study and peak PCWP values were within the normal range (< 20 mmHg) at baseline and study end. Nevertheless, we observed a significant increase in peak PCWP after 24 weeks compared to baseline, which may reflect a positive effect of increased flow through the pulmonary circuit.

Peak VO_2_ is an important prognostic marker in PAH and defines exercise limitations that result from abnormalities in the cardiopulmonary circuit. In patients with PAH, VO_2_ is generally reduced and reflects the decreased ability to augment pulmonary and systemic blood flow during exercise.^27^ In addition, as PVR increases, there is a decrease in CO and impaired oxygen delivery to skeletal muscle. Our results show significant improvements in CO consistent with improved flow through the pulmonary circuit and increased peak VO_2._

Systemic oxygen extraction during exercise, reflected by a widening difference in Ca-vO_2_, normally increases threefold during short-term incremental exercise. Our results show a significant decline in Ca-vO_2_ at maximum exercise after ambrisentan treatment. This is currently an active area of investigation in our laboratory.

We also observed improvements among secondary endpoints, including WHO FC, 6MWD, and perceived breathlessness. At baseline, the majority of participants presented with FC 2, indicating a slight limitation of physical activity resulting in undue dyspnea or fatigue. All participants who completed study improved in functional class after six months of treatment, able to perform ordinary physical activity without limitation. Exercise capacity, as assessed by 6MWD, also improved significantly after the treatment period. On average, participants completed the 6MWT with only moderate dyspnea at study end, compared to intense dyspnea at baseline. Notably, it took an average of three months for participants to notice a subjective improvement in their exercise capacity and dyspnea on exertion. This observation builds on our hypothesis that treatment of ePH with ambrisentan results in ongoing reverse remodeling of the pulmonary vascular network, which needs to reach a certain threshold before a difference in functional status is noticed.

## Limitations

While results are promising, important limitations of the present study must be considered. This is a single-center, open-label trial where all patients were referred to our pulmonary vascular disease program based on the results of a prior clinically indicated iCPET. Results should be interpreted with caution in view of the small sample size and the lack of a control group. However, reports such as this can provide valuable information about current clinical practices and can guide the design of randomized controlled trials. End-of-study data were not collected from the eight participants withdrawn due to subjective lack of improvement and subjective worsening dyspnea on exertion. However, those patients transitioned to a PDE-5 inhibitor then realized improvement, which supports our hypothesis that patients with ePH represent an early and potentially readily treatable disease state.

Open label designs also raise concern for the introduction of bias in the results as it is difficult to control for benefits from study enrollment that are unrelated to study treatment, such as more frequent follow-up and increased disease awareness. While bias may have influenced secondary endpoints, it is very unlikely to have had any effect on invasively measured cardiopulmonary hemodynamics. A single-center, single-operator study design allows for an accurate comparison of data from baseline to study end and minimizes the potential for performance bias. While the need for a larger randomized controlled trial is justified, the prevalence of ePH and ability to perform iCPETs at other institutions pose considerable difficulties.

Of note, a recently published study has proposed a revised definition of ePH which is based on age-specific upper limits of normal for maximum exercise pulmonary hemodynamics.^9^ In the current study, we chose to continue participant recruitment under original inclusion criteria which included a lower upper limit of normal for PVR max (<1.0 WU), for enrollment consistency. Only two of 22 participants included in the analysis of this study had a low baseline PVRmax, which would not affect overall trends and significant changes reported.

## Conclusion

Patients with ePH may provide a unique window into the pathogenesis of PAH, as an early phase of disease with an abnormal pulmonary vascular response to exercise. Our findings suggest that treatment of ePH with ambrisentan results in improved cardiopulmonary hemodynamics and functional status over a six-month period, on average. Treatment of ePH may prevent the progression of vascular remodeling and development of established PAH. Further study of ePH treatment is warranted, especially with regards to disease progression, functional capacity, and quality of life.

## Supplementary Material

Supplementary material

## References

[1] Badesch DB Champion HC Sanchez MA Diagnosis and assessment of pulmonary arterial hypertension. J Am Coll Cardiol 2009; 54(Suppl. 1): S55–66. 19555859 10.1016/j.jacc.2009.04.011

[2] Hoeper MM Bogaard HJ Condliffe R Definitions and diagnosis of pulmonary hypertension. J Am Coll Cardiol 2013; 62(Suppl. 25): D42–50. 24355641 10.1016/j.jacc.2013.10.032

[3] Tolle JJ Waxman AB Van Horn TL Exercise-induced pulmonary arterial hypertension. Circulation 2008; 118(21): 2183–2189. 18981305 10.1161/CIRCULATIONAHA.108.787101 PMC2767322

[4] Fowler RM Maiorana AJ Jenkins SC Implications of exercise-induced pulmonary arterial hypertension. Med Sci Sports Exerc 2011; 43(6): 983–989. 21085040 10.1249/MSS.0b013e318204cdac

[5] Waxman AB Bennett C Janocha AJ Abnormal transpulmonary metabolite flux in exercise induced pulmonary arterial vasculopathy. Am J Respir Crit Care Med 2016; 193: A2909.

[6] Herve P Lau EM Sitbon O Criteria for diagnosis of exercise pulmonary hypertension. Eur Respir J 2015; 46(3): 728–737. 26022955 10.1183/09031936.00021915

[7] Naeije R Vanderpool R Dhakal BP Exercise-induced pulmonary hypertension: physiological basis and methodological concerns. Am J Respir Crit Care Med 2013; 187(6): 576–583. 23348976 10.1164/rccm.201211-2090CI PMC3733438

[8] Naeije R Vonk Noordegraaf A Kovacs G Exercise-induced pulmonary hypertension: at last!. Eur Respir J 2015; 46(3): 583–586. 26324684 10.1183/09031936.00061015

[9] Oliveira RK Agarwal M Tracy JA Age-related upper limits of normal for maximum upright exercise pulmonary haemodynamics. Eur Respir J 2016; 47(4): 1179–1188. 26677941 10.1183/13993003.01307-2015

[10] Oliveira RK Waxman AB Agarwal M Pulmonary haemodynamics during recovery from maximum incremental cycling exercise. Eur Respir J 2016; 48(1): 158–167. 27126692 10.1183/13993003.00023-2016

[11] Benza RL Miller DP Barst RJ An evaluation of long-term survival from time of diagnosis in pulmonary arterial hypertension from the REVEAL Registry. Chest 2012; 142(2): 448–456. 22281797 10.1378/chest.11-1460

[12] Badesch DB Raskob GE Elliott CG Pulmonary arterial hypertension: baseline characteristics from the REVEAL Registry. Chest 2010; 137(2): 376–387. 19837821 10.1378/chest.09-1140

[13] Kovacs G Avian A Tscherner M Characterization of patients with borderline pulmonary arterial pressure. Chest 2014; 146(6): 1486–1493. 25451346 10.1378/chest.14-0194

[14] Condliffe R Kiely DG Peacock AJ Connective tissue disease-associated pulmonary arterial hypertension in the modern treatment era. Am J Respir Crit Care Med 2009; 179(2): 151–157. 18931333 10.1164/rccm.200806-953OC

[15] Kovacs G Maier R Aberer E Borderline pulmonary arterial pressure is associated with decreased exercise capacity in scleroderma. Am J Respir Crit Care Med 2009; 180(9): 881–886. 19679693 10.1164/rccm.200904-0563OC

[16] Saggar R Khanna D Furst DE Exercise-induced pulmonary hypertension associated with systemic sclerosis: four distinct entities. Arthritis Rheum 2010; 62(12): 3741–3750. 20722025 10.1002/art.27695 PMC7065301

[17] Steen V Chou M Shanmugam V Exercise-induced pulmonary arterial hypertension in patients with systemic sclerosis. Chest 2008; 134(1): 146–151. 18403670 10.1378/chest.07-2324

[18] Whyte K Hoette S Herve P The association between resting and mild-to-moderate exercise pulmonary artery pressure. Eur Respir J 2012; 39(2): 313–318. 21737562 10.1183/09031936.00019911

[19] Saggar R Khanna D Shapiro S Brief report: effect of ambrisentan treatment on exercise-induced pulmonary hypertension in systemic sclerosis: a prospective single-center, open-label pilot study. Arthritis Rheum 2012; 64(12): 4072–4077. 22777623 10.1002/art.34614

[20] Park MH Ramani GV Kop WJ Exercise-uncovered pulmonary arterial hypertension and pharmacologic therapy: Clinical benefits. J Heart Lung Transplant 2010; 29(2): 2. 10.1016/j.healun.2009.10.010 20113912

[21] Kovacs G Maier R Aberer E Pulmonary arterial hypertension therapy may be safe and effective in patients with systemic sclerosis and borderline pulmonary artery pressure. Arthritis Rheum 2012; 64(4): 1257–1262. 22127844 10.1002/art.33460

[22] Yagi S Akaike M Iwase T Bosentan ameliorated exercise-induced pulmonary arterial hypertension complicated with systemic sclerosis. Intern Med 2010; 49(21): 2309–2312. 21048365 10.2169/internalmedicine.49.3812

[23] Galie N Olschewski H Oudiz RJ Ambrisentan for the treatment of pulmonary arterial hypertension: results of the ambrisentan in pulmonary arterial hypertension, randomized, double-blind, placebo-controlled, multicenter, efficacy (ARIES) study 1 and 2. Circulation 2008; 117(23): 3010–3019. 18506008 10.1161/CIRCULATIONAHA.107.742510

[24] Ben-Yehuda O Pizzuti D Brown A Long-term hepatic safety of ambrisentan in patients with pulmonary arterial hypertension. J Am Coll Cardiol 2012; 60(1): 80–81. 22578922 10.1016/j.jacc.2012.03.025

[25] Maron BA Cockrill BA Waxman AB The invasive cardiopulmonary exercise test. Circulation 2013; 127(10): 1157–1164. 23479667 10.1161/CIRCULATIONAHA.112.104463

[26] Berry NC Manyoo A Oldham WM Protocol for exercise hemodynamic assessment: performing an invasive cardiopulmonary exercise test in clinical practice. Pulm Circ 2015; 5(4): 610–618. 26697168 10.1086/683815 PMC4671735

[27] Waxman AB Exercise physiology and pulmonary arterial hypertension. Prog Cardiovasc Dis 2012; 55(2): 172–179. 23009913 10.1016/j.pcad.2012.07.003

[28] Lankhaar JW Westerhof N Faes TJ Quantification of right ventricular afterload in patients with and without pulmonary hypertension. Am J Physiol Heart Circ Physiol 2006; 291(4): H1731–1737. 16699074 10.1152/ajpheart.00336.2006

[29] Lankhaar JW Westerhof N Faes TJ Pulmonary vascular resistance and compliance stay inversely related during treatment of pulmonary hypertension. Eur Heart J 2008; 29(13): 1688–1695. 18349027 10.1093/eurheartj/ehn103

[30] Malenfant S Potus F Mainguy V Impaired skeletal muscle oxygenation and exercise tolerance in pulmonary hypertension. Med Sci Sports Exerc 2015; 47(11): 2273–2282. 25970662 10.1249/MSS.0000000000000696

[31] Tolle J Waxman A Systrom D Impaired systemic oxygen extraction at maximum exercise in pulmonary hypertension. Med Sci Sports Exerc 2008; 40(1): 3–8. 18091026 10.1249/mss.0b013e318159d1b8

