# Supplementary material for: Open label study of ambrisentan in patients with exercise pulmonary hypertension
Source: Pulm Circ. 2017 May 12;7(2):531–8. doi: 10.1177/2045893217709024 (PMC5467947; doi:10.1177/2045893217709024)
Supplement: Supplementary material [file PUL709024_supplementary_material.pdf]

Online Supplemental Materials

Open label study of ambrisentan in patients with exercise pulmonary hypertension

STUDY OBJECTIVE

The objective of this 6-month study was to evaluate the effects of ambrisentan administered orally on exercise capacity utilizing iCPET in patients with ePH. The proportion of subjects who achieved categorical improvement was assessed by a change from Baseline to Week 24.

SECONDARY OBJECTIVES

Secondary objectives include investigating the effect of ambrisentan on the proportion of subjects who achieved categorical improvement in the 6MWT and WHO functional class assessment. The proportion of subjects who achieved categorical improvement was assessed by a change from Baseline to Week 24.

STUDY DESIGN

This was a single-center, open-label trial. The study consisted of Screening, Baseline and Treatment Phases. Patients who provided appropriate informed consent and who met all inclusion/exclusion criteria during the Screening Phase entered the Baseline Phase. All patients were recruited after having had a clinically indicated iCPET performed for the purpose of evaluating unexplained dyspnea on exertion. If results of iCPET were consistent with ePH, patients were recruited into the study.

Immediately after baseline measurements were obtained and all inclusion/exclusion criteria were met, ambrisentan therapy was initiated.

During the 6-month Treatment Phase, safety, exercise capacity and clinical symptoms of PAH were assessed at specified times. Patients were withdrawn from the study after completion of final study assessments after 6 months, and continued on ambrisentan or transitioned to other therapy, at the discretion of the Investigator.

At the end of the 6-month Treatment Phase an iCPET and Investigator-assessed WHO functional class were assessed. End of treatment 6MWD and Borg dyspnea score were assessed at Week 20.

INCLUSION CRITERIA

A subject was included in the study only if he/she met all of the following inclusion criteria:

1. The subject provided written informed consent before the commencement of any study related procedure.
2. The subject was 18 years of age or older.
3. If a female subject of child-bearing potential, the subject agreed to use 2 forms of contraceptive therapy, including at least 1 barrier method, throughout the

study and follow-up. (Women who were surgically sterile or those post-menopausal for at least 2 years were not considered to be of childbearing potential.)

4. The subject had findings of ePH on an iCPET performed within 6 months of Screening, and was WHO functional class I-III.
5. The subject had an LVEF ≥ 55%, obtained by any appropriate method (i.e., ECHO, radionuclide imaging, or cardiac catheterization) within 6 months of Screening.
6. The subject was taking a stable concomitant medication regimen for at least 4 weeks prior to enrollment in the study that was not expected to change during the study period and follow-up. Changes in diuretic and/or nitrate therapy as needed during the study period were acceptable.

EXCLUSION CRITERIA

A subject meeting any of the following criteria was not eligible to participate in the study:

1. The subject had clinically significant psychiatric, addictive, neurologic disease or any other condition that, in the Investigator's opinion, would compromise his/her ability to give informed consent, participate fully in this study, or prevent adherence to the requirements of the study protocol.
2. The subject had evidence of unstable cardiovascular disease including intermittent atrial fibrillation or unstable angina within the 4 weeks prior to Screening.
3. The subject had diagnosis of exercise heart failure with preserved ejection fraction (previously diastolic dysfunction).
4. The subject had amyloidosis, hypertrophic obstructive cardiomyopathy, restrictive cardiomyopathy, or constrictive pericarditis.
5. The subject had a history of myocardial infarction, coronary artery bypass graft surgery, or percutaneous cardiac intervention within 3 months of Screening.
6. The subject had clinically significant valvular heart disease, in the opinion of the Investigator.
7. The subject had a history of cerebrovascular accident or transient ischemic attack within 3 months of Screening.
8. Subject had a serum ALT or AST lab value that was greater than 1.5x ULN prior to Baseline visit.
9. Subject had discontinued other ERA treatment (e.g. bosentan) for any adverse experience.
10. The subject had, in the opinion of the Investigator, a dependence on alcohol.
11. The subject had, in the opinion of the Investigator, a dependence on illicit drugs.
12. The subject had anemia, defined as Hgb below 10.0 g/dL.
13. The subject had exercise tolerance limited by noncardiac causes (e.g., exercise-induced asthma, chronic obstructive pulmonary disease, interstitial lung disease, malignancy, obesity, musculoskeletal disorder).
14. The subject had uncontrolled systemic hypertension defined as a resting blood pressure of ≥ 140/90 mmHg if on no treatment for systemic hypertension or

≥ 160/90 mmHg if on ≥ 2 systemic hypertension medications. For subjects who were receiving treatment for diabetes mellitus, uncontrolled systemic hypertension was defined as ≥ 130/80 mmHg.

15. The subject had the presence, or history, of malignancy that required significant medical intervention within the preceding 3 months and/or was likely to result in death within the next 2 years.
16. The subject had chronic renal impairment or renal insufficiency defined by a serum creatinine ≥ 2.5 mg/dL and/or the requirement for dialysis.
17. The subject was lactating, breastfeeding, or pregnant.
18. The subject received any chronic prostacyclin, prostacyclin analogue, ET receptor antagonist, or phosphodiesterase (PDE) inhibitor therapy within the 30 days prior to study entry. The use of PDE inhibitors "as needed" for erectile dysfunction was acceptable as long as the subject was not dosed within 24 hours of an efficacy assessment.
19. The subject had a documented allergy to Lidocaine.
20. Had received any investigational medication within 30 days prior to the start of this study or scheduled to receive another investigational drug during the course of this study.

STUDY SCHEDULE

Prior to conducting any study-related procedures, written informed consent was obtained from the subject. Participation in the study was voluntary. The nature of the study was fully explained to each subject during the informed consent process and the subject had the opportunity to ask questions. An informed consent form was signed by the subject and the Investigator performing the consent discussion. A copy of the signed informed consent form was given to the subject.

Screening and Baseline Evaluations – Day 1 Visit

Screening procedures were completed prior to initiation of treatment. Following successful completion of the Screening Phase and meeting all non-invasive inclusion/exclusion criteria, each patient entered the Baseline Phase.

- Obtain written informed consent, before conducting any study-related procedure
- Screen for inclusion/exclusion criteria
- Review of historical iCPET (Appendix A) and RHC results to ensure subject meets the etiology-specific inclusion criteria for right heart, pulmonary arterial, and pulmonary capillary wedge pressure hemodynamics
    - All historical hemodynamic data must have been assessed within 6 months of the Screening visit
- Record subject's medical history
- Perform a physical examination
- Record body weight and height
- Record vital sign measurements pre dose and 1 hour post dose

- Collect information about the current use of medications
- Perform a 12-lead ECG
- Collect chemistry, hematology, and BNP panels; coagulation analysis for subjects receiving warfarin (Appendix F)
- Collect blood sample for serum beta human chorionic gonadotropin (β-hCG) pregnancy test on women of childbearing potential
- Administer the 6MWT (Appendix B)
- Administer Borg dyspnea scale (Appendix C)
- Assess WHO functional class (Appendix D)
- Dispense study drug and instruct subject to return all ambrisentan container(s) at the next visit so compliance can be evaluated
- Collect AE information post dosing, if necessary (Appendix E)
- Instruct subject that they will take ambrisentan on the mornings of follow-up clinic visits

Treatment Phase – Subsequent Monthly Study Visits
The Treatment Phase began on Study Day 1 immediately after completion of all baseline assessments.

All patients were started at 5mg of ambrisentan by mouth every day. Patients continued on 5mg daily for 4 weeks. At the Week 4 visit, patients were increased to 10mg, if tolerating the 5mg dose, and remained on 10mg for the duration of the study.
If the patient experienced intolerable adverse events on 10mg, the dose was decreased to 5mg and the patient remained on 5mg for the duration of the study.

Upon discharge from the clinic, the patient was provided with a supply of ambrisentan. The Investigator assured the adequacy of outpatient healthcare and may have involved the patient's local physician or other health service.

The following assessments were performed at the Week 4, 8, 12, 16, and 20 visits for all subjects:

- Record date and time of the previous ambrisentan dose
- Record vital sign measurements prior to the morning dose of ambrisentan
- Administer the 6MWT prior to taking dose of ambrisentan
- Administer Borg dyspnea scale
- Assess WHO functional class
- Collect chemistry and hematology panels
- Collect serum pregnancy test for women of childbearing potential
- Record current use of medications
- Assess and record AE's
- Account for all dispensed drug from the previous visit
- Dispense ambrisentan and instruct the subject to return all ambrisentan container(s) at the next visit so compliance can be evaluated

Adverse experiences (Appendix F) were recorded in the patient's CRF during all phases of the study. In addition, each patient had telephone access to the Investigator for immediate consultation should any problems arise. Depending on the nature and severity of the event, the patient, local physician or Investigator cared for any adverse experiences occurring outside the hospital. The Investigator instructed each patient as to the types of events requiring self, local physician, or Investigator attention. The Investigator was responsible for responding to and recording the adverse experiences appropriately. Any adverse experiences, including clinically significant abnormal lab values, were followed to resolution or until no longer considered clinically significant.

End of Study – Week 24 (± 4 Days) or Early Termination

At the end of 24 weeks, all patients returned to the hospital to be evaluated. Prior to the completion of any assessments, the Investigator determined whether the subject was able to safely perform the EOS iCPET. Subjects remained on study drug until all Week 24 assessments were complete.

- Record date and time of the previous ambrisentan dose
- Record vital sign measurements prior to the morning dose of ambrisentan
- Perform a physical examination
- Assess WHO functional class
- Perform a 12-lead ECG
- Collect chemistry, hematology, and BNP panels; coagulation analysis for subjects receiving warfarin
- Collect serum pregnancy test for women of childbearing potential
- Record current use of medications
- Assess and record AE's
- Account for all dispensed drug from the previous visit
- Perform iCPET (not performed during Early Termination visit)

All subjects on warfarin were instructed to stop warfarin 5 days before the visit. On the morning of the visit a PT/INR was collected. The results of the PT/INR needed to be ≤ 1.5 for RHC and arterial catheter insertion. If the PT/INR was > 1.5 the procedure was cancelled until the PT/INR was ≤ 1.5.

Upon completion of all Week 24 assessments, patients were dismissed from the study. Patients remained on ambrisentan therapy, or other therapy was initiated after completion of the study, at the discretion of the Investigator.

Subject Withdrawal Criteria

Subjects reserved the right to discontinue the trial at any time for any reason. Subjects who withdrew from the study had an Early Termination visit performed at the time of the subject's study withdrawal, or at the soonest possible date. Assessments conducted at the Early Termination visit are summarized above.

Subject Discontinuation Criteria

A subject was discontinued from the study in response to any of the following situations:

1. The Investigator or attending physician decided that the subject should be discontinued.
2. The subject was required to discontinue study drug, for any reason, for > 7 consecutive days.
3. The subject developed any condition that alters exercise tolerance, limits ambulation, or otherwise results in a contraindication to or an inability to perform the 6MWT.
4. The subject developed acute coronary syndrome.
5. The subject developed an ST segment elevation myocardial infarction.
6. The subject developed significant anemia (Hgb concentration fell below 8.0 g/dL) that was unresponsive to medical intervention (e.g., iron and vitamin supplements, blood transfusion).
7. The subject required additional chronic (≥ 14 consecutive days) HF treatment during the study period (i.e., the addition of angiotensin receptor blockers, beta blockers).
8. The subject developed elevations in liver enzyme values >1.5x ULN.
9. The subject became pregnant during the study period.

28-Day Post Study Safety Follow-Up Visit (± 7 Days)

This visit may be conducted via telephone.

- Assess and record AE's
- Record current use of medications

STATISTICAL EVALUATION

As this was a small, open-label study, statistics are descriptive. A total of 30 patients with a diagnosis of ePH were enrolled. Based on clinical findings in our patient population and from published literature, an average pre-treatment mPAP of 33 mmHg and post-treatment mPAP of 28 mmHg were used (SD = 7.5) to determine an appropriate sample size. A 5 percent significance level and 80 percent power yield a sample size of 18 subjects in pre- and post-treatment groups. A 15% improvement in any of the outcome measures is considered clinically significant. For all efficacy endpoints, data from study assessments during the treatment phase will be compared to baseline assessments. As each subject served as his/her own control, one-sample t-test was utilized under the normality assumption. If normality failed, an appropriate non-parametric rank test (e.g., Wilcoxon) was utilized. Data from each of the primary and secondary efficacy variables are summarized in tabular form. Continuous data will have means and standard deviations. Ordinal or categorical data will have frequencies and percentages tabulated. Graphical presentations are also displayed.

DATA AND SAFETY MONITORING PLAN

The PI was responsible for the safety monitoring of data for this protocol. Safety data was reviewed as the research team obtained information from subjects. This information was obtained at every 4-week follow-up visit or by telephone.

CONFIDENTIALITY

In order to maintain subject privacy, all CRFs, study drug accountability records, study reports, and communications identified subjects by initials and the assigned subject number.

APPENDIX A - INVASIVE CARDIOPULMONARY EXERCISE TEST (ICPET)

Pulmonary Function Tests

1. *Spirometry with Reversibility:* Spirometry testing will be conducted using a spirometer maintained and calibrated according to manufacturer specifications and compliant with current standards established by the American Thoracic Society (ATS). The spirometers must be validated for accuracy prior to subject testing. Short acting inhaled bronchodilators (e.g., albuterol, ipratropium bromide) will be withheld for at least 4 hours prior to testing. A minimum of 3 and maximum of 6 individual forced vital capacity (FVC) maneuvers will be performed in an effort to obtain 3 acceptable (e.g., <5% back-extrapolated volume, no coughing in first second, and reasonable duration of expiration for at least 6 seconds or until a plateau in the volume-time curve is maintained for at least 1 second) and 2 repeatable (largest and second largest FVC within 150 mL, and largest and second largest FEV_1 within 150 mL) efforts (according to current ATS standards).

   Once the pre-bronchodilator assessment is complete, subjects will be asked to use 2 puffs of their own albuterol metered dose inhaler after which the spirometry will be re-assessed in the same manner. The following values will be reported for each series (pre and post) of spirometry:

   - Largest FVC from either of the 2 repeatable maneuvers
   - Largest FEV_1 from either of the 2 repeatable maneuvers
   - Largest FEV_6 from either of the 2 repeatable maneuvers
   - FEV_1/FVC from the largest FEV_1 and FVC reported

2. *Body Plethysmogrophy:* Plethysmogrophy will be done using the whole-body plethysmography maintained and calibrated according to manufacture specifications and complaint with current standards established by the ATS. A volume-displacement plethysmogrophy measures volume changes of the thorax directly. Subjects breathe in and out across the wall of the plethysmograph to room air. The increase in lung volume that occurs during inspiration includes the volume of gas inspired plus the additional volume associated with decompression of thoracic gas volume (TGV) resulting from the fall in intrathoracic pressure necessary to provide a gradient of inspiratory airflow.
3. *Carbon Monoxide Diffusing Capacity:* A test of the diffusing capacity of the lungs for carbon monoxide (DLCO) is one of the most clinically valuable tests of lung function. Standards for DLCO instruments, performance of the test, and calculation of the results are established by the ATS. DLCO measures the ability of the lungs to transfer gas from inhaled air to the red blood cells in pulmonary capillaries.

Hemodynamic Measurements

RHC is performed using local anesthesia at the Brigham and Women's Hospital with heparin bonded double lumen Swan-Ganz Catheters (Baxter Laboratories) inserted percutaneously via the internal jugular vein using ultrasound guidance. The catheter is positioned in the pulmonary artery with pressure monitoring. The catheter tip position

is confirmed using fluoroscopy. A 20-gauge heparin bonded cannula is inserted percutaneously into a radial artery for blood sampling and monitoring of systemic arterial pressure. Pulmonary capillary wedge pressure will be measured, and mean vascular pressures will be calculated based on systolic and diastolic pressure measurements. Heart rate and vascular pressures are monitored continuously. Cardiac output is measured by the Fick method. Systemic and pulmonary vascular resistances are calculated. The acute hemodynamic effects of inhaled nitric oxide delivered at 80ppm at rest are measured.

Physiologic Measurements

All exercise tests are performed at the BWH Cardiopulmonary Exercise Laboratory, using an upright cycle ergometer. A flow-directed, balloon-tipped, pulmonary arterial catheter is inserted using either the right internal-jugular or left subclavian vein approach, and directed into the pulmonary artery. If necessary, fluoroscopic guidance is used. An arterial line is inserted percutaneously into the radial artery using a 20-gauge plastic catheter. Systemic and pulmonary artery pressures are measured continuously on the monitoring system utilized in the BWH Exercise Laboratory. The monitoring system is calibrated before each study. Mean end-expiratory values for right atrial pressure (RAP) and pulmonary artery pressure (PAP) are obtained, in addition to systemic arterial pressure (BP). Three-milliliter samples of systemic and pulmonary artery blood are obtained at rest in triplicate and during each minute of exercise, and are analyzed for PO_2, PCO_2, pH and O_2 saturation (SaO_2), hemoglobin concentration (Hb), and O_2 content (CaO_2) by co-oximetry**.** One-milliliter samples of systemic arterial blood are also analyzed for lactate concentration**.** An additional 7cc of blood is obtained at rest, peak exercise, and one-hour post peak exercise for evaluation of vasoactive mediators, metabolomic profiling, and circulating endothelial cells and progenitor cells.

Pulmonary gas exchange and minute ventilation (V_E) are measured breath-by-breath using a commercially available metabolic cart whose methodology has been previously validated.

Exercise Protocol

Patients complete a trial of incremental exercise to a symptom-limited maximum. The tests are performed with the subject breathing room-air. Two minutes of rest are followed by 2 minutes of unloaded cycling at 40-60 RPM. Work rate is continuously increased using a ramp protocol at 6.25, 12.5, or 25 W/min. Ventilation, pulmonary gas exchange, heart rate (HR), BP, RAP, and PAP are measured continuously while pulmonary capillary wedge pressure (PCWP) and a 12-lead ECG are obtained at rest and each minute of exercise. Three-milliliter blood samples are simultaneously drawn from the radial artery and distal port of the non-wedged pulmonary artery catheter during the last minute of the rest period and during the last 15 seconds of each minute during exercise and one hour following completion of exercise. Ventilatory and hemodynamic parameters are measured for one hour following completion of exercise.

Data Analysis

Resting ventilatory and gas exchange data are obtained from the averaged final 30-second interval of the 2-minute rest period. Exercise ventilatory and gas exchange data are averaged over contiguous 30-second intervals. V_D/V_T and $AaDO_2$ are calculated from the standard formulae.

VO_2max is defined as the highest 30-second averaged VO_2 during the last minute of the symptom-limited exercise test. Predicted values of VO_2max were those of Hansen and colleagues. Cardiac output (Q_t) is calculated from the Fick principle ($Q_t = VO_2/[CaO_2 - CvO_2]$), and stroke volume from Q_t/HR. Predicted maximal Q_t is calculated from predicted VO_2max and an assumed maximal arterial-venous O_2 content difference equivalent to hemoglobin level for healthy untrained subjects. Oxygen delivery (DO_2) is calculated as Q_t*CaO_2. Pulmonary vascular resistance (PVR) is calculated as (PAP – PCWP)/Q_t. Systemic vascular resistance is calculated as (MAP – RAP)/Q_t. The systemic oxygen extraction ratio (SER) is calculated as (CaO_2 – CvO_2)/CaO_2.

At maximum exercise, PAH is defined as PAP$\geq$30mmHg, PCWP$\leq$20mmHg, PVR$\geq$80dynes-sec/cm^5.

APPENDIX B - PROCEDURE FOR SIX-MINUTE WALK EXERCISE TEST AND BORG DYSPNEA SCALE

General Procedures

The area used for the 6MWT should be pre-measured at a minimum of 108 feet (33 meters) in length and at least 6 to 10 feet (2 to 3 meters) in width. The length should be marked with half-yard (0.5 meter) gradations. The area should be well ventilated with air temperature controlled at 20 to 23 degrees C (68 to 76 degrees F).

The tester may be at the starting end of the corridor or at the midpoint of the corridor with a stopwatch. Intermittent rest periods are allowed if the patient can no longer continue. If the patient needs to rest briefly, he/she may stand or sit and then begin again when he/she is sufficiently rested but the clock will continue to run. At the end of six minutes, the tester will call "stop" while simultaneously stopping the watch and then measure the distance walked. The Borg dyspnea scale will be administered.

Instructions to the Patient

Patients will be instructed that the preceding meal should be light. Patients should be told to wear comfortable clothing and sneakers or comfortable walking shoes. The person administering the test will use the following exact dialogue with the patient:

> *"The purpose of this test is to find out how far you can walk in six minutes. You will start from this point and follow the hallway to the marker (e.g. chair) at the end, turn around and walk back. When you arrive back at the starting point you will go back and forth again. You will go back and forth as many times as you can in the six-minute period. You may stop and rest if you need to. Just remain where you are until you can go on again. However, the most important thing about the test is that you cover as much ground as you possibly can during the six minutes. I will tell you the time, and I will let you know when the six minutes are up. When I say STOP, please stand right where you are."*

After these instructions are given to the patient, the person administering the test will then ask:

> *"Do you have any questions about the test?"*
> *"Please explain to me what you are going to do."*

The person administering the test will then start the test by saying the following to the patient:

> *"Are you ready?"*
> *"Start when I say "GO."*

The person administering the test will tell the patient the time at 2 and 4 minutes by saying:

> *"You have completed 2 minutes."*

And then by saying:

> *"You have completed 4 minutes."*

No other instruction or encouragement will be given during the test. Eye contact with the patient should be avoided during the test.

Following the walk, the person administering the test will obtain a rating of dyspnea using the Borg scale.

APPENDIX C - MODIFIED BORG DYSPNEA SCALE

The modified Borg dyspnea scale should be printed on heavy paper (11 inches high, possibly laminated) in 20-point font size. This scale will need to be in an unobstructed line of sight in front of the subject.

The person will use the following dialogue:

> *"I would like to use the following scale to indicate the maximal shortness of breath you had during the walk test (indicate the Borg dyspnea scale). If there was no shortness of breath at all you would point to 0; if the shortness of breath was not very great you would choose from 0.5 to 2; if you were somewhat more short of breath you would select 3; and if the breathing was getting very difficult, you would choose 4 to 9, depending on just how hard it was; 10 represent the greatest shortness of breath you have ever experienced in your life, and if you feel more short of breath than you have ever been in you life before, choose a number greater that 10 that represents how short of breath you feel. If one of the numbers does not exactly represent how short of breath you are, then you can choose a fraction between. For example, if you had shortness of breath somewhere between 4 and 5, you could choose 4 ½."*

0	Nothing at all
0.5	Very, very slight (just noticeable)
1	Very slight
2	Slight (light)
3	Moderate
4	Somewhat severe
5	Severe (heavy)
6	
7	Very severe
8	
9	
10	Very, very severe (maximal)

APPENDIX D - WHO FUNCTIONAL CLASS ASSESSMENT

	WHO Functional Assessment
Class I	Subjects with cardiac disease but without resulting limitation of physical activity. Ordinary physical activity does not cause undue fatigue, palpitation, dyspnea, or anginal pain.
Class II	Subjects with cardiac disease resulting in slight limitation of physical activity. They are comfortable at rest. Ordinary physical activity results in fatigue, palpitation, dyspnea, or anginal pain.
Class III	Subjects with cardiac disease resulting in marked limitation of physical activity. They are comfortable at rest. Less than ordinary activity causes fatigue, palpitation, dyspnea, or anginal pain.
Class IV	Subjects with cardiac disease resulting in inability to carry on any physical activity without discomfort. Symptoms of heart failure or the anginal syndrome may be present even at rest. If any physical activity is undertaken, discomfort is increased.

APPENDIX E - ADVERSE EVENT REPORTING

The PI or a designated member of his staff will probe each patient for any adverse experiences, which may have occurred. The Investigator should always ask the same question when conducting the verbal probe in order to ensure uniformity between patients. The investigator should ask:

“How are you doing (feeling)?”

Based on the patient’s response to this question, the investigator should ask additional questions relevant to the specific complaint such as:

“How severe is/was the symptom?”
“How often did the symptom occur?”
“How long did the symptom last?”

Using definitions that follow, the Investigator will then: (1) rate the intensity and seriousness of the adverse experience, (2) estimate the causality of the adverse experience to, and (3) note actions taken to counteract the adverse experience.

Definitions of Intensity, Seriousness, Causality, and Countermeasures

Intensity: An assessment of the relative intensity (severity) of an adverse experience is based on the Investigator’s clinical judgment. The maximum intensity encountered during the evaluation period should be checked. The assessment of intensity should be independent of the assessment of the seriousness of the adverse experience.

Seriousness: A serious adverse experience is one that represents an actual or potential significant hazard. This includes, but is not limited to, an experience that is fatal, life-threatening, permanently or severely disabling, requires or prolongs inpatient hospitalization, is a congenital abnormality (offspring of patient) or is medically significant (important medical events that may not result in death, be life-threatening, or require hospitalization may be considered an SAE when, based upon appropriate medical judgment, they may jeopardize the patient and may require medical or surgical intervention to prevent one of the outcomes listed in this definition).

Causality: The Investigator makes an estimate of causality between a specified adverse experience and the study medication. Definitions of the categories follow:

- Not reasonably attributable – it is unlikely that the adverse experience was caused by the test agent
- Possibly attributable – the adverse experience may have been caused by the test agent
- Reasonably attributable – it is likely that the test agent caused the adverse experience

Countermeasures:

- Test agent dose modification
    - None – there was no alteration in either the dose or regimen of the test agent
    - Test agent dose adjusted – the dose or regimen of the test agent was altered or administration of the test agent was stopped temporarily

    - Test agent stopped permanently – administration of the test agent was stopped permanently and not restarted
- Other action taken – refers only to concomitant therapy or other actions for management of the adverse experience, not to alternative treatment of the disease/condition under investigation

APPENDIX F – LABORATORY ASSESSMENTS

**Chemistry**	Sodium
	Potassium
	Chloride
	Bicarbonate
	Urea Nitrogen
	Glucose
	Creatinine
	Calcium
	Albumin
	Alkaline phosphatase
	Total bilirubin
	Total protein
	AST
	ALT
**Hematology**	CBC with platelets
**Coagulation**	PT/INR
**Other**	NT-pro-BNP
